# Free water diffusion MRI and executive function with a speed component in healthy aging

**DOI:** 10.1016/j.neuroimage.2022.119303

**Published:** 2022-05-12

**Authors:** Martin Berger, Lukas Pirpamer, Edith Hofer, Stefan Ropele, Marco Duering, Benno Gesierich, Ofer Pasternak, Christian Enzinger, Reinhold Schmidt, Marisa Koini

**Affiliations:** a Department of Neurology, Division of Neurogeriatrics, Medical University of Graz, Auenbruggerplatz 22, Graz 8036, Austria; b Institute for Medical Informatics, Statistics and Documentation, Medical University of Graz, Graz, Austria; c Medical Image Analysis Center (MIAC AG) and Department of Biomedical Engineering, University of Basel, Basel, Switzerland; d Institute for Stroke and Dementia Research (ISD), University Hospital, Munich, Germany; e Departments of Psychiatry and Radiology, Brigham and Women’s Hospital, Harvard Medical School, Boston, MA, USA; f Department of Neurology, Medical University of Graz, Graz, Austria

**Keywords:** Free water diffusion, Cognitive speed, Corpus callosum, Fornix, Executive functions, Aging

## Abstract

Extracellular free water (FW) increases are suggested to better provide pathophysiological information in brain aging than conventional biomarkers such as fractional anisotropy. The aim of the present study was to determine the relationship between conventional biomarkers, FW in white matter hyperintensities (WMH), FW in normal appearing white matter (NAWM) and in white matter tracts and executive functions (EF) with a speed component in elderly persons.

We examined 226 healthy elderly participants (median age 69.83 years, IQR: 56.99–74.42) who underwent brain MRI and neuropsychological examination. FW in WMH and in NAWM as well as FW corrected diffusion metrics and measures derived from conventional MRI (white matter hyperintensities, brain volume, lacunes) were used in partial correlation (adjusted for age) to assess their correlation with EF with a speed component. Random forest analysis was used to assess the relative importance of these variables as determinants. Lastly, linear regression analyses of FW in white matter tracts corrected for risk factors of cognitive and white matter deterioration, were used to examine the role of specific tracts on EF with a speed component, which were then ranked with random forest regression.

Partial correlation analyses revealed that almost all imaging metrics showed a significant association with EF with a speed component (*r* = −0.213 – 0.266). Random forest regression highlighted FW in WMH and in NAWM as most important among all diffusion and structural MRI metrics. The fornix (R^2^=0.421, *p* = 0.018) and the corpus callosum (genu (*R*^2^ = 0.418, *p* = 0.021), prefrontal (*R*^2^ = 0.416, *p* = 0.026), premotor (*R*^2^ = 0.418, *p* = 0.021)) were associated with EF with a speed component in tract based regression analyses and had highest variables importance.

In a normal aging population FW in WMH and NAWM is more closely related to EF with a speed component than standard DTI and brain structural measures. Higher amounts of FW in the fornix and the frontal part of the corpus callosum leads to deteriorating EF with a speed component.

## 1. Introduction

Brain aging is often associated with small vessel disease (SVD) which manifests with both focal and widespread microstructural tissue changes detectable by MRI (Shindo et al., 2020). Frequently, SVD is accompanied by deteriorating cognitive functions particularly including frontal-executive deficits manifesting in reduced cognitive speed, poorer working memory and reduced set-shifting ability (Sachdev et al., 2014). Diffusion tensor imaging (DTI) is able to detect microstructural tissue alterations by quantifying the diffusion properties of water (Bennett et al., 2010; Zhang et al., 2010). Changes in DTI measures, such as, reduction of fractional anisotropy (FA) and increases of mean diffusivity (MD), radial and axial diffusivity (RD and AD) were reported in normal appearing white matter in subjects with cerebral SVD and were more closely related to cognitive dysfunction than lesions that are visible in structural MRI (Duering et al., 2018; Edde et al., 2020). Recently, the free water (FW) diffusion MRI model has gained particular interest in SVD, but also in other diseases of the brain (Andica et al., 2019; Bergamino et al., 2017; Duering et al., 2018; Ofori et al., 2019; Yang et al., 2019). The model determines a FW compartment that represents water molecules that are not restricted or directed and thus represents the extracellular space (Chad et al., 2018a; Pasternak et al., 2009) while the tissue compartment represents intra- and extracellular water that is restricted by physical barriers like axonal membranes and myelin (Duering et al., 2018). Duering and co-workers (Duering et al., 2018) have shown that in genetic and sporadic forms of cerebral SVD, an increase of FW is more closely related to the clinical status of patients than DTI measures representing the tissue compartment. Moreover, results from the Alzheimer’s Disease Neuroimaging Initiative showed that free water imaging of the white matter can serve as an early stage marker for Alzheimer’s disease (Dumont et al., 2019) and similar findings have been reported in Parkinson’s disease (Burciu et al., 2017). Recently, a study in forty-seven older adults described that extracellular FW within white matter increases also with normal aging, and that the amount of FW is indirectly related to fluid cognitive functions (Gullett et al., 2020).

We hypothesize that FW in WMH and in NAWM are more strongly related to EF with a speed component than conventional DTI and MRI parameters in a large community-dwelling cohort of elderly subjects free of stroke and dementia. We therefore evaluated the relative importance of the FW metric as a determinant of EF with a speed component compared to standard structural MRI measures and conventional DTI metrics by use of a random forest analysis. Based on the crucial importance of frontal-subcortical circuits in mediating EF with a speed component we also hypothesized that the effects of FW changes on cognition, if any, are most pronounced in white matter tracts of the frontal lobe than in other lobes. We examined the importance of the FW metric relative to other DTI and volumetric measures in 42 white matter tracts. A random forest analysis was used to rank the white matter tracts according to their importance for EF with a speed component. This is, to the best of our knowledge, the first study in neurologically normal community-dwelling subjects examining the role of the FW content in the global white matter and in specific white matter tracts EF with a speed component relative to other DTI and structural MRI measures.

## 2. Methods

### 2.1. Participants

The Austrian Stroke Prevention Study (ASPS) is a prospective single-center community-based study established in 1991 on the cerebral effects of vascular risk factors in the normal aged (age range: 44–90 years) population of the city of Graz, Austria. ASPS family (ASPS-Fam) represents an extension of the ASPS (Schmidt et al., 1999, 1994), where between 2006 and 2013, study participants of the ASPS and their first-degree relatives were recruited. Inclusion criteria were no history of previous stroke or dementia and a normal neurologic examination. A total of 419 individuals of which 381 had MRI from 169 families were included in the study. The number of members per family ranged from 2 to 6. The entire cohort underwent a thorough diagnostic workup including clinical history, laboratory evaluation, cognitive testing, and an extended vascular risk factor assessment. All individuals underwent MRI, except for 26 who had contraindications. Two-hundred and sixty-seven subjects had a diffusion weighted MR scan, of which 238 passed the quality check. Of these, 226 were also tested for EF with a speed component and formed the current investigational cohort.

The study protocol was reviewed and approved by the ethics committee of the Medical University of Graz, Austria. All subjects provided written informed consent to participate in this study.

### 2.2. Risk factors

The following determinants were included in the study as possible confounders in the association between DTI metrics and EF with a speed component: age, sex, hypertension (definition: history of hypertension with repeated blood pressure readings ≥ 140/90 mmHg, treatment of hypertension, or readings at the examination exceeding the limit), diabetes (definition: history or current treatment of diabetes or fasting blood glucose level at the examination > 126 mg/dl), hypercholesterolemia (definition: history or current treatment of hypercholesterolemia, total cholesterol at examination > 200 mg/dl or low-density lipoprotein at examination > 130 mg/dl), body mass index (BMI) and smoking (definition: current smoking yes/no).

### 2.3. Neuropsychological examination

The study protocol included a thorough test battery examining cognitive speed, global cognitive functioning, verbal and visual memory, and executive function. The tests employed have been widely used in the German-speaking area and were always applied in the same order and under same laboratory conditions. For a detailed description see Freudenberger et al. (2016), Ghadery et al. (2015). The timed tests assessed were (1) a computerized complex reaction time measured with the Wiener Reaktionsgerät (cRT) (Wiener Reaktionsgeraet, 1991), part of the Schuhfried psychological test battery; (2) time to finish the Trail Making Test-B (TMT-B) (Army Individual Test, 1944) and; (3) the assembly score of the Purdue Pegboard Test (PPT-a) (Tiffin and Asher, 1948). The TMT-A has not been part of the neuropsychological assessment protocol. The three neuropsychological assessment scores were factor analysed using explorative principal component analysis with Varimax (orthogonal) rotation to examine whether the three assessments load on the same factor. The analysis yielded a single factor explaining a total of 60.7% of the variance for the entire set of variables. The bivariate correlation matrix yielded significant correlations > 0.3 among the three assessments (cRT and TMT-B: *r* = 0.346, *p* < 0.01; cRT and PPT-a *r* = 0.357, *p* < 0.01; TMT-B and PPT-a: *r* = 0.520, *p* < 0.001), a Kaiser-Meyer-Olkin measure of 0.638 and a significant Bartlett’s test (Chi-square (3) = 109.860, *p* < 0.001). Communalities were cRT = 0.488, TMT-*B =* 0.662 and PPT-*a* = 0.671. Factor loadings without rotation were cRT = 0.699, TMT-*B* = 0.813 and PPT-*a* = 0.819.We then calculated a composite score of EF with a speed component variable by averaging the z-transformed scores.

### 2.4. Acquisition of MRI data

Magnetic resonance imaging data were acquired on a 3T whole-body MR system (TimTrio; Siemens Healthcare, Erlangen, Germany) using a 12-channel head coil. For diffusion tensor imaging (DTI) we used a 2D echo planar imaging (EPI) diffusion sequence with 12 directions, *b*-value = 1000s/mm^2^ , TR/TE = 6700/95 ms, resolution = 2 × 2 × 2.5 mm, matrix = 128 × 128px, number of slices = 50, parallel imaging (GRAPPA = 2), with one b0 image and four repetitions. The study protocol included a high-resolution T1 weighted 3D sequence with magnetization prepared rapid gradient echo (MPRAGE) with whole brain coverage (TR = 1900 ms, TE = 2.19 ms, TI = 900 ms, flip angle = 9°, isotropic resolution of 1 mm) for assessing brain volume and for tissue segmentation. To assess white matter lesion load, we performed a T2w-FLAIR sequence (TR = 10,000 ms, TE = 69 ms, TI = 2500 ms, TI = 800/1100 ms (1.5/3 T), number of slices = 40, slice thickness = 3 mm, in-plane resolution = 0.9 × 0.9 mm^2^ ).

### 2.5. Preprocessing and image analysis

Diffusion data were processed using the Functional Magnetic Resonance Imaging of the Brain (FMRIB) Diffusion Toolbox (FDT) (Behrens et al., 2003) included in the FMRIB Software Library (FSL, version 6.0.1) (Smith et al., 2004). For each participant we first extracted a brain mask on the un-weighted diffusion image (*b*-value = 0) using FSL-bet (part of FSL), following motion and eddy-current correction by FSL’s eddy for each subject (Andersson and Sotiropoulos, 2016).

After visual inspection, FW-corrected and uncorrected diffusion tensors were calculated for each voxel as previously described (Pasternak et al., 2009). In brief, the diffusion signal of each voxel was fitted to a two-compartment model. The tissue compartment was modeled by a diffusion tensor, and the FW compartment was modeled as isotropic diffusion tensor with a constant diffusion coefficient of water at 37 °C. The resulting FW values, ranging from 0 to 1, represent the relative contribution of the FW compartment in each voxel. The FW-corrected tensor represents the microstructure of the tissue compartment without the influence of FW. Tissue compartment measures (denoted by the subscript t) including mean diffusivity (MDt), fractional anisotropy (FAt), radial diffusivity (RDt) and axial diffusivity (ADt) were calculated by tensor-decomposition of the FW-corrected tensor.

Regional median-DTI measures were assessed by registering the T1 and FLAIR sequences to the diffusion space and by applying the transformation-matrices to the binary WM and WMH masks. The registration was performed with six degrees of freedom and a mutual-information cost function implemented in FSL-flirt (Jenkinson and Smith, 2001). To ensure that the white matter (WM) mask was not affected by partial volume effects, caused by the registration into the less-resolved diffusion space, we used a robust threshold of 90% and inspected each registration manually. For each subject an individual normal appearing white matter (NAWM) mask was computed by subtracting the lesions (WMH masks) from the FreeSurfer segmented WM mask. Assessment of regional and global brain volume as well as regional segmentation including a mask for global WM, was performed on the T1-weighted MPRAGE sequence using the FreeSurfer image analysis suite, which is documented and freely available for download online. Median values of FW-corrected axial diffusivity (ADt), mean diffusivity (MDt), radial diffusivity (RDt) and fractional anisotropy (FAt) were assessed in NAWM and in WMH using fslstats.

White matter tracts were assessed using FreeSurfer-TRACULA (TRActs Constrained by UnderLying Anatomy) (version 7.2.2, freely available online https://surfer.nmr.mgh.harvard.edu) which reconstructs 42 major white matter pathways using global probabilistic tractography with anatomical neighborhood priors (https://surfer.nmr.mgh.harvard.edu/fswiki/Tracula). As input data of the TRACULA pipeline we used the eddy-current and FW corrected diffusion weighted MRI data. We performed a visual inspection of all tracts and we excluded 21 of the 226 subjects due to tract-reconstruction failures or registration-misalignments. Tracts of both hemispheres were merged. Averaged FW values were assessed in each merged region (*n* = 25). Tracts included are: arcuate fasciculus, acoustic radiation, anterior thalamic radiation, cingulum bundle dorsal and ventral, corticospinal tract, extreme capsule, frontal aslant tract, fornix, inferior and middle longitudinal fasciculus, optic radiation, superior longitudinal fasciculus I, II and III, uncinated fasciculus, anterior commissure, and the corpus callosum (body central, body parietal, body prefrontal, body premotor, body temporal, genu, rostrum, splenium).

To estimate the volume of WMH and to assess median-DTI measures in WMH, binary masks were drawn on the T2w-FLAIR sequence by an experienced rater. WMH maps were generated by using a custom-written Interactive Data Language program as described previously (Plummer, 1992). Thereby, the rater identified each lesion slice by slice by marking a point inside each lesion and the algorithm automatically drew the borders of the lesion. In case of misclassifications, the rater modified the lesion masks manually. WMH volume was obtained by fslstats (part of FSL), evaluated in the native FLAIR-space. Afterwards WMH volume was normalized by intracranial volume. Brain tissue volume, normalized for subject head size, was estimated with SIENAX (Smith et al., 2002, 2001), part of FSL (Smith et al., 2004). As a further potential determinant of EF with a speed component, presence of lacunes were also included. Lacunes were defined as focal lesions that involved the basal ganglia, internal capsule, thalamus, or brainstem, not exceeding a diameter of 15 mm.

### 2.6. Statistical analyses

Statistical analysis was performed using SPSS (version 26, SPSS Inc., Chicago, IL) and the statistical software R (version 4.0.2; R: a language and environment for statistical computing, Vienna, Austria). A two-sided *p*-value < 0.05 was considered to be statistically significant. The following imaging variables were included in the analyses: FW in NAWM and WMH, FW-corrected values of FA, MD, AD and RD in NAWM and WMH, normalized brain volume, normalized WMH volume and the presence of lacunes. After visual inspection of scatterplots between the imaging variables and EF with a speed component, WMH volume was ln-transformed and FW in NAWM was transformed exponentially to achieve a linear association between the dependent and the explanatory variables of the multiple linear regression. Thereafter, we performed partial correlation between imaging parameters and EF with a speed component adjusted for age. To determine the importance of imaging variables as determinants of EF with a speed component, we calculated a random forest regression. Random forest assesses the mean prediction of each individual variable while at the same time accounting for all other variables. Using the R package ‘party’ (version 1.3–5 (Strobl et al., 2007) 1001 inference trees with unbiased variable selection were calculated using 4 and 5 randomly selected variables for each split (unbiased resampling scheme). From these trees conditional permutation importance, using the “mean decrease in accuracy” principle as importance measure for each variable, together with a 95% confidence interval from 400 repetitions, was calculated. This approach was chosen to account for the intercorrelation between predictor variables (multicollinearity), and to identify the variables with highest importance. A high conditional variable importance reflects the impact of the predictor variable on the outcome, when “controlling” for the other variables. Hence, the variable importance mimics the behavior of a partial correlation or linear regression coefficients (Strobl et al., 2008, 2007). Percentages given are calculated within the random forest model relative to the other variables in the model.

To assess the association between specific supratentorial white matter tracts and EF with a speed component linear regression analyses adjusted for the risk factors age, sex, hypertension, diabetes, hypercholesterolemia, BMI and smoking were calculated. Similarly, the subsequent random forest analysis served for the identification and illustration of the variable importance of the predictors.

### 2.7. Data availability

Requests for data should be made to marisa.koini@medunigraz.at.

## 3. Results

**Fig. 1.**
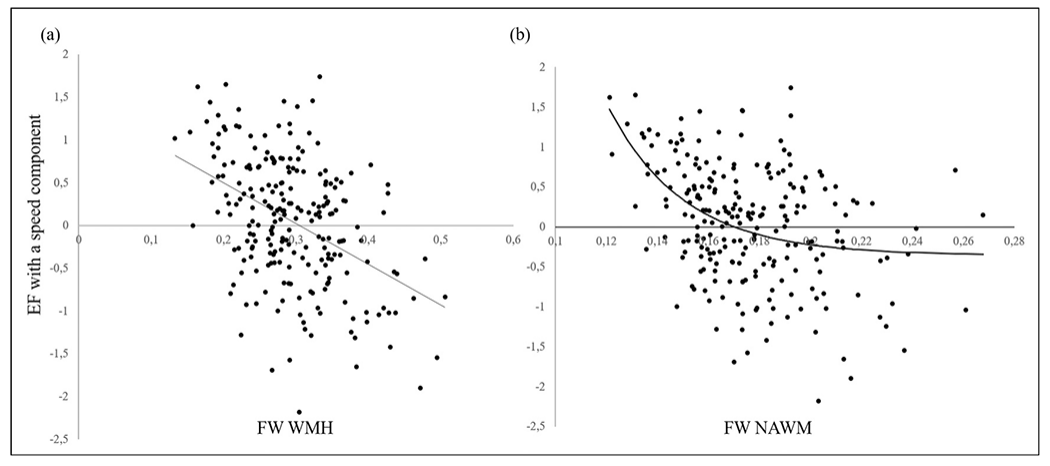
Scatterplots and fit lines of FW in WMH (a) and in NAWM (b) (x-axis) and EF with a speed component (y-axis).

Overall, 226 individuals were included. For demographics and risk factors see Table 1. Lacunes were present in 22 individuals. For characteristics of the imaging parameters see Table 2. As can be seen in Fig. 1, the association between FW in WMH and EF with a speed component was linear, whereas the association between FW in NAWM and EF with a speed component was exponential. Consequently, FW in NAWM was transformed for further calculations.

Partial correlation, adjusting for age, showed that almost all diffusion MRI measures and lacunes were significantly related with EF with a speed component (Table 3) while no significant association existed for WMH volume and brain volume (Table 3).

**Fig. 2.**
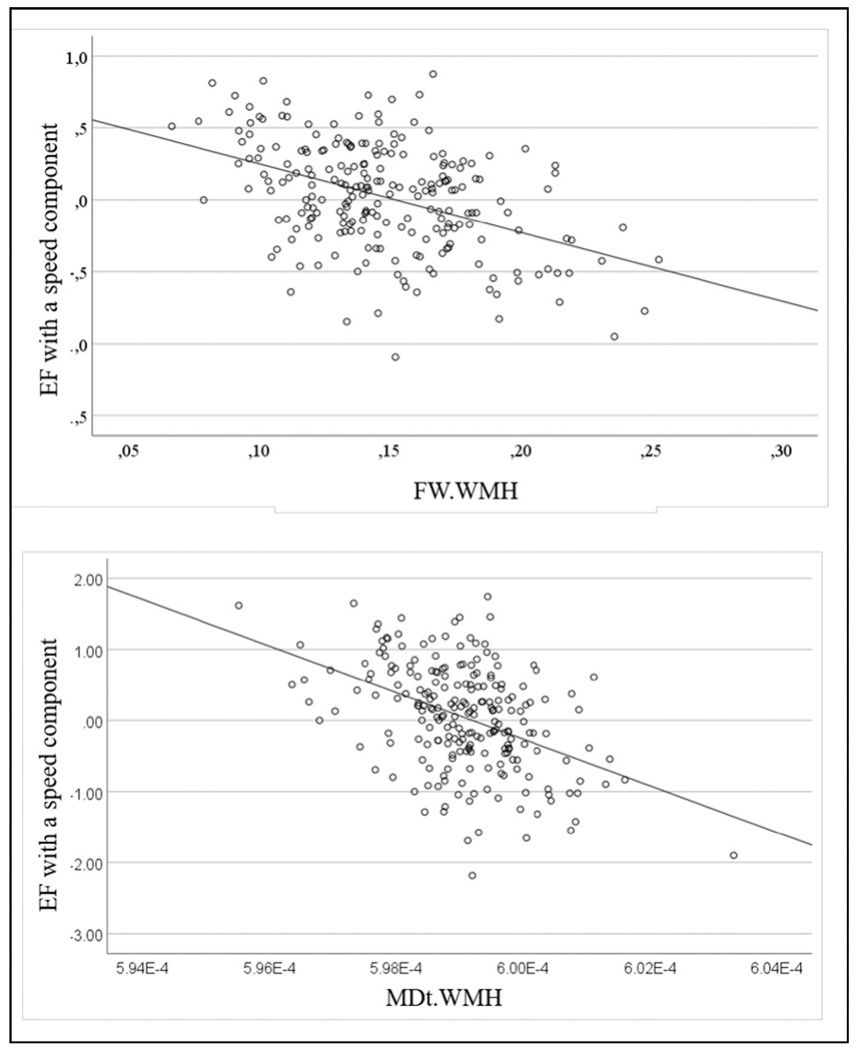
Scatterplots showing an indirect relationship between two exemplarily chosen DTI measures in white matter hyperintensities (WMH) and cognitive functioning. The upper panel demonstrates the relationship between FW and the lower panel between MDt and results on EF with a speed component testing.

Fig. 2 shows two examples of the relationship between DTI measures and cognitive test results. As can be seen from this figure there was an indirect association between FW and FAt in white matter hyperintensities and EF with a speed component. Similar relationships existed for DTI measures in the NAWM and cognitive performance.

**Fig. 3.**
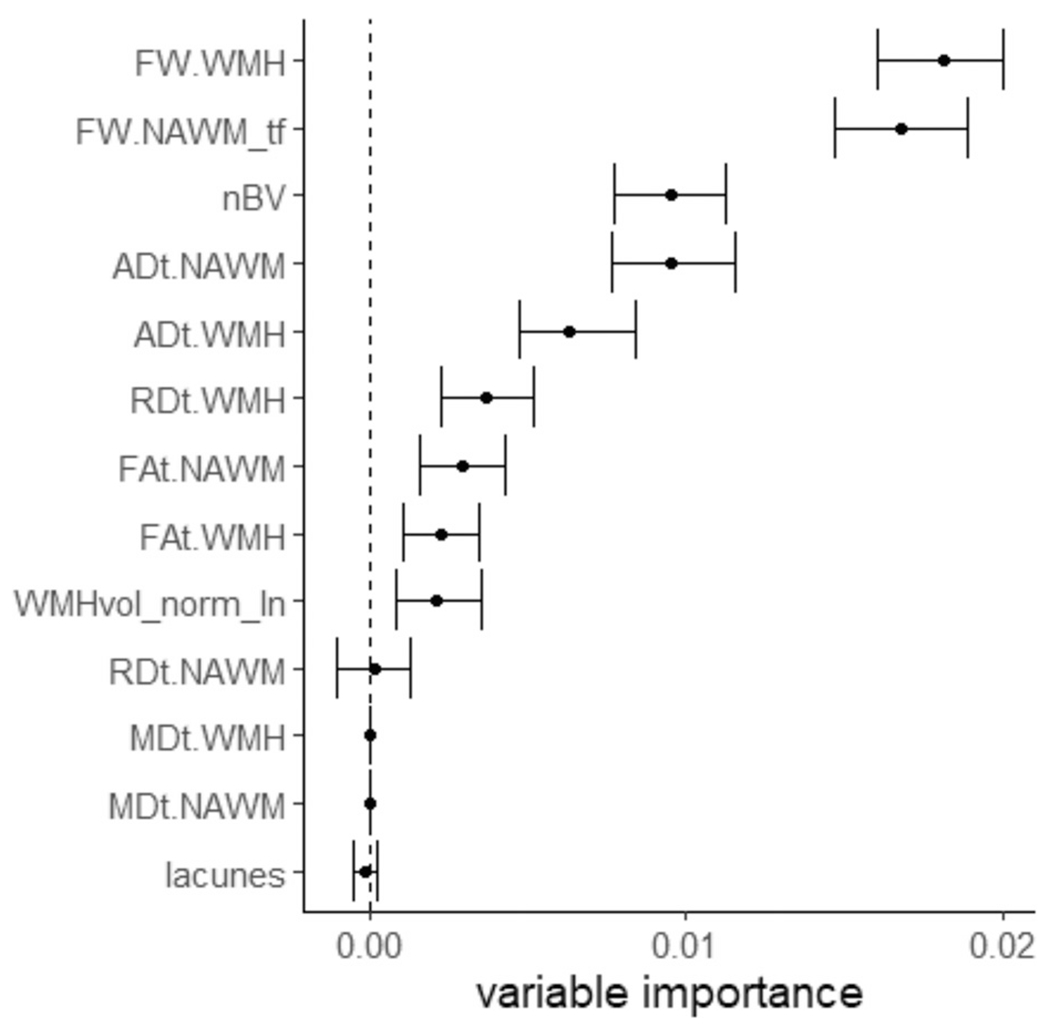
Random forest regression analysis: Association between brain MRI measures and EF with a speed component. Free water in WMH and in NAWM were shown to have highest variable importance for EF with a speed component in a random forest regression. Abbreviations: FW = free water, WMH = white matter hyperintensities, NAWM = normal appearing white matter, tf = transformed, *t* = suffix for free water corrected tissue, AD = axial diffusivity, nBV = normalized brain volume, FA = fractional anisotropy, RD = radial diffusivity, WMHvol_norm_ln = normalized and log-transformed WMH volume, MD = mean diffusivity.

Random forest regression showed that FW in WMH (Mean = 0.018, IQR = 0.016–0.020) and in NAWM (Mean = 0.016, IQR = 0.015–0.019) had the highest conditional variable importance for EF with a speed component (Fig. 3). Together the contribution of the two variables explain 48.8% importance for EF with a speed component while accounting for intercorrelations (multicollinearity). The other FW-corrected diffusion parameters showed lower importance for EF with a speed component. Among macrostrucutral brain alterations normalized brain volume showed highest importance (Mean = 0.010, IQR = 0.008–0.011).

**Fig. 4.**
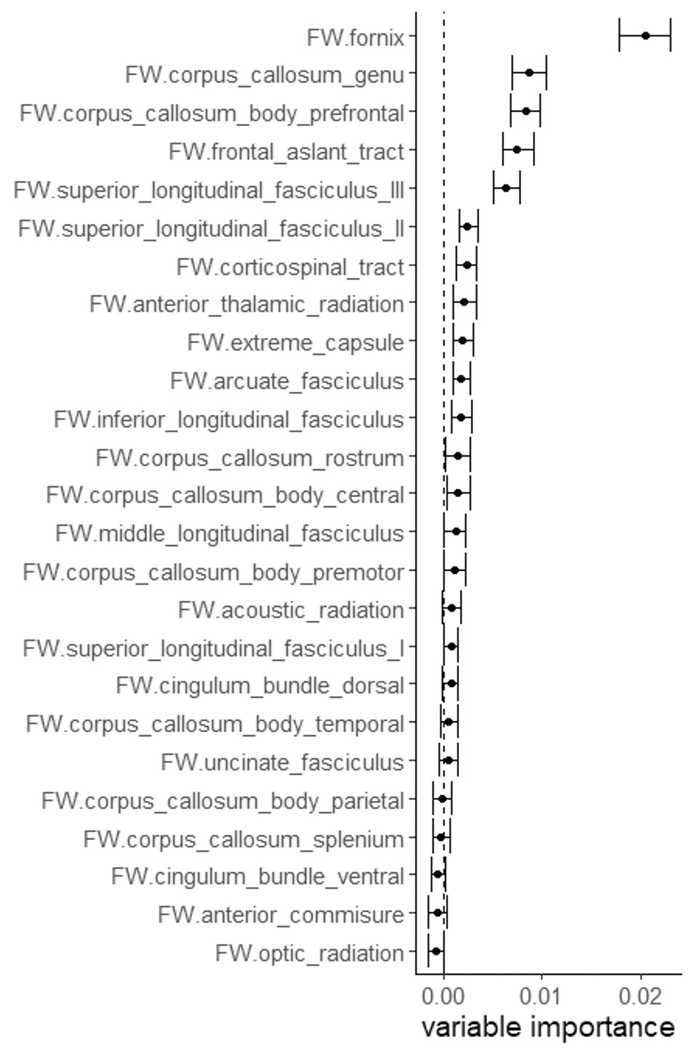
Random forest regression analysis. Association between FW in specific white matter tracts and EF with a speed component. FW in the fornix and the corpus callosum (genu, prefrontal part of the body) showed highest variable importance for EF with a speed component.

The relative importance of FW for EF with a speed component was then assessed in a tract-specific manner. Linear regression analyses including age, sex, hypertension, diabetes, hypercholesterolemia, BMI and smoking were used to regress EF with a speed component on each white matter tract. Results revealed an association with the fornix and the corpus callosum (body prefrontal, body premotor, genu) **(**Table 4). No other examined tract has been associated with EF with a speed component. The random forest regression ranked the fornix, the genu and the prefrontal part of the body of the corpus callosum as most important (Fig. 4).

## 4. Discussion

In a cohort of community-dwelling subjects without stroke and dementia extracellular FW in WMH and NAWM are the most important determinants of EF with a speed component. The FW scalar outperformed other micro- and macrostructural brain measures such as FAt, MDt, RDt, ADt, WMH volume and brain volume when controlling for age. Similar findings have been reported in patients with genetic forms of small vessel disease (Duering et al., 2018). Our study for the first time shows similar association in a community-dwelling cohort without symptoms or signs of cerebrovascular disease or dementia. The superior role of the FW signal as a determinant of EF with a speed component suggests that an increase of extracellular water in the brain’s white matter is connected with cognitive dysfunction in elderly individuals, while alterations in the tissue compartment, reflecting fiber structure are less important. Our study extends previous work by assessing FW in the white matter not only globally but also in a tract-based analysis. We found significant negative associations between FW and EF with a speed component in the fornix and the corpus callosum. The association between the fornix with EF with a speed component is in line with previous literature (Burzynska et al., 2017).The association between FW in the corpus callosum and EF with a speed component indicates that age related loss of interhemispheric white matter integrity also determines cognitive slowing (Banich and Belger, 1990).

Several studies have already corroborated the value of FW as a predictor of cognitive function with aging (Archer et al., 2020; Edde et al., 2020; Maillard et al., 2019; Reas et al., 2018). Maillard et al. (2019) reported FW, corrected for hippocampal volume, to be the strongest predictor for cognitive dysfunction and cognitive decline, in a longitudinal study on 224 subjects with cognitive performance between 0 and 3 on the Clinical Dementia Rating Scale (Hughes et al., 1982). Similar to our results, FW outperformed the conventional diffusion parameters. Our data indicate that metrics of FW are better suited to determine brain’s white matter microstructure than conventional DTI parameters or structural metrics to explain aging-related frontal-executive deterioration (Chad et al., 2018b). This notion gets corroborated by two recent studies demonstrating an increase of FW in the white matter (Dumont et al., 2019) and the hippocampus (Ofori et al., 2019) paralleling the continuum from normal cognitive function to Mild Cognitive Impairment towards Alzheimer’s disease. These results remained significant when controlling for brain atrophy. We extent this research by describing the association between FW and EF with a speed component in a large normal elderly cohort including separate indices for FW in WMH and NAWM.

The underlying pathophysiological mechanism leading to higher extracellular fluid content in subjects with cognitive dysfunction is still unknown. Dumont et al. (2019) assumed that FW can be used to understand the role of inflammation-associated edema in Alzheimer’s disease. Other substrates besides neuroinflammation such as vascular changes and neurodegeneration with subsequent CSF diffusion and perfusion effects, atrophy and a microscopic degradation have also been implied (Maillard et al., 2019). There is indeed evidence that elevated blood pressure and arterial stiffness may trigger a pathophysiological cascade leading to increased FW content and subtle WM degeneration (Maillard et al., 2017). The study authors suggested that increasing arterial stiffness leads to transmitted pressure waves on high-resistance cerebral small vessels. This can lead to endothelial injury, subtle breakdown of the blood brain barrier and fluid transudation as the basis of FW increase. Conceivably, the fluid passing the blood brain barrier then introduces toxic serum cytokines or reduces the clearance of interstitial toxins, leading to a breakdown of WM microstructure (Maillard et al., 2019, 2017). The involvement of vascular alterations gains support by the finding that diffusion alterations are largely determined by SVD. Finsterwalder and colleagues compared individuals with Alzheimer’s disease and SVD, including genetically defined samples and found that markers for SVD were strongly associated with diffusion measures (Finsterwalder et al., 2020).

The fornix has in the past been shown to be associated with aspects of memory that are important for encoding, consolidation and recall of declarative memory (Thomas et al., 2011) but also with cognitive speed (Burzynska et al., 2017) working memory, motor performance and problem solving (Zahr et al., 2009). These results indicate that the increase of FW in the fornix in healthy elderly is not restricted to memory tasks, but rather is involved in a broader spectrum of cognitive abilities.

Age related decline of the integrity of global cerebral white matter has been observed. However, the disruption of white matter networks, i.e. specific regional white matter tracts critical for cognitive integrity might be more specific for cognitive deterioration in healthy aging. Hence, they yield the potential for a more precise picture of the association between microstructural white matter alterations and cognitive changes. Age related decline of the integrity of white matter have been reported to be more pronounced in frontal than in posterior brain regions (Davis et al., 2009; Sullivan et al., 2006; Zahr et al., 2009), and in more superior compared to inferior regions (Sullivan et al., 2010). These differences in white matter deterioration reflect a diverging vulnerability of the white matter. However, on the other hand the variation of deterioration in specific tracts might not be entirely specific to individual tracts, but instead can also be reflected by more global effects, common to the brain as a whole (Johnson et al., 2015). Using principal component analysis in older adults (71—73 years) on each of several DTI measures from eight white matter tracts, Penke et al. (2010) found that a single factor explained approximately 45% of the variance in each measure. Also, many of the individual tracts loaded highly on each individual factor. Further, two of these global components were correlated with information processing speed. The authors concluded that these results underscore that individual differences in white matter integrity are shared globally throughout the brain and that they are related to processing speed. Consequently, we decided to examine microstructural white matter changes and its association with executive function with a speed component in a whole brain approach and in a tract-specific approach. By doing so, we found that whole brain white matter integrity and also the integrity of the fornix and the anterior parts of the corpus callosum were involved in cognitive functioning of our study participants.

Our findings indicate that anterior segments of the corpus callosum are particularly important for EF with a speed component. This result is in line with two studies in healthy aging (Kennedy and Raz, 2009) and Mild Cognitive Impairment (Raghavan et al., 2020) both reporting especially the genu of the corpus callosum to be associated with and predictive for cognitive change over time. One limitation of the study is its cross-sectional design. However, we examined the association of FW and EF with a speed component in a large cohort of community-dwelling subjects with a thorough clinical work-up. While our results match with current literature, future studies should consider the acquisition of multi-shell diffusion data which may improve the accuracy of the free-water model fit, and may allow distinguishing further biological components that contribute to the increase in free water. Another limitation of the free water metric is that we did not account for biases due to molecules in rapid random motion, such as the intravoxel incoherent motion (IVIM) of blood as suggested by Rydhög et al. (2017). The authors emphasize the importance for the correction of water molecules perfusing in randomly oriented capillary networks. The suggested three-compartment model in their scientific work was described to disentangle the effects of free water diffusion and perfusion, which is of clinical importance when trying to ascribe different pathologies to free water alterations. Future studies should take care of IVIM.A weakness of our study is that the DTI protocol was limited to twelve directions. This likely hampered the robustness of results. Yet, the protocol was performed with four repetitions. Importantly, Basser and coworkers have shown that multiple averages of diffusion tensor reconstructions provide comparable data to a protocol with 30- or more directions (Basser et al., 1994). Additionally, the negative association between FAt in WMH and executive function with a speed component was an unexpected result that warrants further investigation.

Our study has shown that in elderly without overt cognitive impairment FW is a strong MRI determinant of cognitive slowing/EF. Longitudinal studies assessing the rate of change of FW with advancing age and its predictive value for cognitive dysfunction are warranted. The pathophysiological basis of FW increase in the aging brain needs to be determined.

## Figures and Tables

**Table 1:** Demographics and risk factors of the cohort.

age (median (IQR))	69.83 (56.99–74.42)
sex (f/m)	133 / 93
hypertension (yes)	143
diabetes (yes)	24
hypercholesterolemia (yes)	175
Body mass index (mean (SD))	26.1 (4.4)
current smoker (yes)	29

**Table 2:** Characteristics of the imaging parameters.

	mean	SD	min	max
FW.NAWM	4.90E-02	3.06E-01	−3.40E-01	1.47E+00
FW.WMH	2.95E-01	6.80E-02	1.33E-01	5.06E-01
ADt.NAWM	9.37E-04	1.63E-05	8.37E-04	9.77E-04
ADt.WMH	9.28E-04	5.54E-05	7.94E-04	1.18E-03
FAt.NAWM	5.33E-01	1.63E-02	4.42E-01	5.67E-01
FAt.WMH	4.89E-01	5.18E-02	3.67E-01	6.94E-01
MDt.NAWM	5.97E-04	9.18E-07	5.94E-04	5.99E-04
MDt.WMH	5.99E-04	9.94E-07	5.96E-04	6.03E-04
RDt.NAWM	4.04E-04	8.11E-06	3.81E-04	4.39E-04
RDt.WMH	4.32E-04	2.36E-05	3.18E-04	4.95E-04
WMH volume*	−5.80E+00	1.05E+00	−9.29E+00	−2.51E+00
nBV	1.49E+06	8.18E+04	1.21E+06	1.72E+06

Abbreviations: FW = free water, NAWM = normal appearing white matter, WMH = white matter hyperintensity, AD = axial diffusivity, FA = fractional anisotropy, MD = mean diffusivity, RD = radial diffusivity; *t* = suffix for free water corrected tissue, nBV = normalized brain volume;

* normalized and ln-transformed.

**Table 3:** Partial correlation between imaging variables and EF with a speed component, adjusted for age.

	r	*p*-value
FW.NAWM*	−0.141	0.036
FW.WMH	−0.171	0.010
MDt.NAWM	−0.058	0.385
MDt.WMH	−0.213	0.001
FAt.NAWM	0.261	<0.001
FAt.WMH	−0.169	0.011
ADt.NAWM	0.266	<0.001
ADt.WMH	−0.199	0.003
RDt.NAWM	−0.196	0.003
RDt.WMH	0.178	0.007
WMH volume**	−0.105	0.116
brain volume (normalized)	0.083	0.213
lacunes^***^	−2.020	0.045

Abbreviation: *r* = correlation coefficient, FW = free water, NAWM = normal appearing white matter, WMH = white matter hyperintensities, MD = mean diffusivity, FA = fractional anisotropy, AD = axial diffusivity, RD = radial diffusivity, *t* = suffix for free water corrected tissue,

* exponentially transformed;

** normalized and ln-transformed;

*** between group *t*-test (presented are T-score and p-value).

**Table 4:** *Significant linear regression results of EF with a speed component regressed on FW in specific white matter tracts.* All analysis are corrected for age and the risk factors hypertension, diabetes, hypercholesterolemia, BMI and smoking. Results are uncorrected for multiple comparisons.

Variable	*B*	Std. error	beta	*p*	VIF	*R*^2^ change	*R* ^2^
age	−0.034	0.004	−0.51	<0.001	1.533	0.017	0.421
hypertension	−0.052	0.09	−0.034	0.565	1.142		
diabetes	−0.19	0.133	−0.081	0.154	1.051		
hypercholesterolemia	−0.161	0.097	−0.093	0.098	1.044		
sex	−0.204	0.083	−0.137	0.015	1.032		
BMI	−0.01	0.01	−0.058	0.326	1.137		
current smoker	−0.132	0.13	−0.059	0.31	1.127		
fornix	−0.567	0.237	−0.154	0.018	1.371		
age	−0.035	0.004	−0.529	<0.001	1.471	0.015	0.416
hypertension	−0.023	0.089	−0.015	0.794	1.149		
diabetes	−0.236	0.132	−0.1	0.075	1.044		
hypercholesterolemia	−0.133	0.097	−0.077	0.168	1.046		
sex	−0.169	0.083	−0.114	0.043	1.05		
BMI	−0.006	0.01	−0.037	0.526	1.137		
current smoker	−0.145	0.126	−0.067	0.25	1.135		
corpus callosum body prefrontal	−2.973	1.329	−0.141	0.026	1.327		
age	−0.035	0.004	−0.538	<0.001	1.374	0.016	0.418
hypertension	−0.012	0.089	−0.008	0.89	1.158		
diabetes	−0.25	0.132	−0.106	0.06	1.049		
hypercholesterolemia	−0.152	0.096	−0.088	0.115	1.042		
sex	−0.161	0.083	−0.109	0.054	1.06		
BMI	−0.006	0.01	−0.038	0.515	1.137		
current smoker	−0.157	0.125	−0.073	0.21	1.125		
corpus callosum body premotor	−3.27	1.402	−0.142	0.021	1.252		
age	−0.035	0.004	−0.528	<0.001	1.449	0.016	0.418
hypertension	−0.025	0.089	−0.016	0.778	1.148		
diabetes	−0.231	0.132	−0.098	0.081	1.043		
hypercholesterolemia	−0.125	0.097	−0.072	0.198	1.053		
sex	−0.17	0.083	−0.114	0.042	1.048		
BMI	−0.005	0.01	−0.03	0.612	1.138		
current smoker	−0.145	0.126	−0.067	0.251	1.134		
corpus callosum genu	−2.996	1.286	−0.146	0.021	1.321		

Abbreviations: *B* = regression coefficient, Std. error = standard error, VIF = variance inflation factor, *R^2^* change = variance explained by the specific tract.

## Data Availability

Requests for data should be made to marisa.koini@medunigraz.at.
